# Monthly Motivational Interview Counseling and Nicotine Replacement Therapy for Smoking Parents of Pediatric Patients: A Randomized Controlled Trial

**DOI:** 10.3389/fped.2022.798351

**Published:** 2022-04-13

**Authors:** Siyu Dai, Michael Ho Ming Chan, Richard Kin Ting Kam, Albert Martin Li, Chun Ting Au, Kate Ching-Ching Chan

**Affiliations:** ^1^Department of Pediatrics, Faculty of Medicine, The Chinese University of Hong Kong, Hong Kong, Hong Kong SAR, China; ^2^School of Clinical Medicine, Hangzhou Normal University, Hangzhou, China; ^3^Department of Chemical Pathology, Prince of Wales Hospital, Hong Kong, Hong Kong SAR, China

**Keywords:** child, clinical settings, environmental tobacco smoke, health hazards, parental, smoke-free, smoking reduction, urine cotinine

## Abstract

**Background:**

Parental smoking is the dominant source of passive smoke exposure in the pediatric population. The current randomized controlled trial (RCT) study aimed to evaluate the effectiveness of a multi-component smoking reduction intervention in parental smoking reduction and children's environmental tobacco smoke exposure reduction in clinical settings.

**Methods:**

A single-blinded, 6-month randomized controlled trial recruited smoking parents (*N* = 210) of children who attended the pediatric wards or clinics at the Prince of Wales Hospital. Participants allocated to the intervention group (*n* = 105) received monthly motivational interviews on smoking reduction with emphasis on health hazards related to children's passive smoke exposure, 8-week nicotine replacement therapy, and referral to smoking cessation service if the parents preferred. The control group (*n* = 105) received simple verbal advice on smoking cessation. Primary outcomes were parental urine cotinine validated and self-reported ≥50% smoking reduction rates at 6 months.

**Results:**

Smoking parents in the intervention group had significantly more biochemically validated ≥50% smoking reduction than the control: 27.1 vs. 10.0% (OR = 3.34, 95% CI: 1.16–9.62, *P* = 0.02). The rate of self-reported ≥50% smoking reduction was also significantly higher in the intervention group than the control: 51.9 vs. 20.2% (OR = 4.40, 95% CI: 2.38–8.12, *P* < *0.0*01). For secondary outcomes, the rate of parental self-reported smoking cessation was higher in the intervention arm: 10.5 vs. 1.0% (OR = 12.17, 95% CI: 1.54–96.07, *P* < *0.0*01), however, no differences were detected in biochemically validated cessation and changes in children's passive smoke exposure between the groups.

**Conclusion:**

Monthly smoking reduction counseling together with nicotine replacement therapy is more effective than simple verbal cessation advice in the smoking reduction for parents of pediatric patients. However, this study did not demonstrate differences in smoking cessation or reduction in children's passive smoke exposure with a 6-month follow-up. Achievement of a smoke-free environment remains challenging.

**Trial Registration:**

Clinicaltrials.gov, identifier: NCT03879889.

## Introduction

The prevalence of environmental tobacco smoke (ETS) exposure in children is ~40% globally and in Hong Kong (1–4). ETS exposure accounts for about 1% of annual worldwide mortality, and 28% of the deaths belong to the pediatric age group (1). The impact of ETS on lung function and childhood lung diseases is well-known (5–7). Children exposed to ETS are also more likely to have a severe influenza infection, vascular disease, and exhibit higher rates of health service utilization (8–11).

Hong Kong implemented the public smoking ban policy in the year 2007. However, there are concerns that comprehensive smoke-free legislation without strong support for cessation could displace smoking into homes, increasing children's passive smoke exposure (12, 13). Reducing parental smoking remains the key to reduce children's exposure (14–17). Pediatricians and healthcare professionals have an important role in screening for the use of tobacco and ETS exposure and providing guidance and referral to reduce children's ETS exposure (18, 19). Furthermore, public awareness of ETS-related hazards remains suboptimal, especially for third-hand smoke (THS) which is the chemical residuals of tobacco smoke that cling to surfaces after the tobacco product is extinguished. It is important to deliver those relevant information to the public and particularly to smoking parents (19–21).

Encountering smoking parents during their children's healthcare visits serves as a golden opportunity to intervene (18, 19). However, there is a lack of randomized controlled trial (RCT) evidence supporting interventions for smoking parents of pediatric patients in clinical settings (15, 22). A community study demonstrated that proactive telephone counseling is an effective aid to promote smoking cessation among parents (23). Unfortunately, its effect on children's ETS exposure was not evaluated (23). Furthermore, study design using biochemical validations with a longer follow-up is recommended for ETS reduction intervention for children (22).

Importantly, unlike smokers who actively seek help for cessation, the majority of smoking parents are not prepared to quit (23). A comprehensive review suggests that reduction-to-quit interventions may be more effective when pharmacotherapy is used as an aid (24). Smoking reduction is an option to provide an intermediate step for complete cessation (16, 25, 26). Studies have shown that people who reduce their smoking are ultimately more likely to quit than people who do not (27–29). Thus, studies to investigate the effectiveness of smoking reduction intervention in parental tobacco use are needed. Moreover, emphasizing benefits to child health as an incentive may further motivate parents to change their smoking behaviors (16).

In the current RCT, a multi-component intervention was evaluated for its effectiveness on smoking reduction in parents of our pediatric patients. Our primary aims were to compare the parental urine cotinine validated and self-reported smoking reduction rates between the intervention and control groups to test the intervention's effectiveness in smoking reduction. Secondary aims were to test the intervention's effectiveness in smoking cessation and in children's ETS reduction.

## Methods

**Figure 1:**
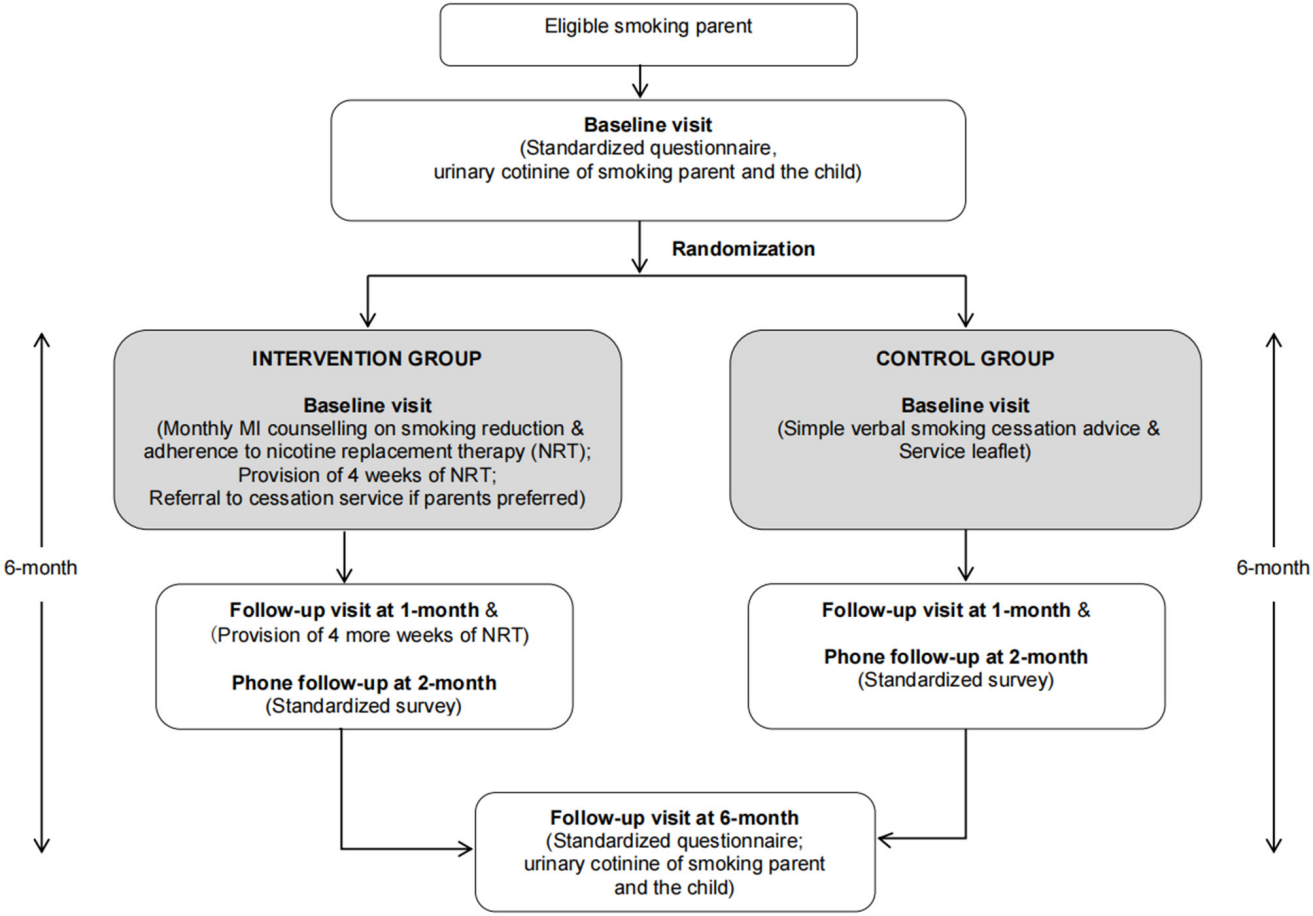
Flow diagram of the study design.

This 6-month RCT was carried out from January 2017 to July 2019. The flow diagram of the study design is shown in Figure 1. Recruited parents were randomly assigned to either intervention [monthly motivational interview (MI) counseling on smoking reduction and 8 weeks of nicotine replacement therapy (NRT)] or control group (simple verbal smoking cessation advice and service leaflet) at a 1:1 ratio. From each family, only one child (our pediatric patient) was recruited. If both parents of the family smoked, both of them were invited and randomized to the same group.

### Ethics Approval

This study was conducted in accordance with the Declaration of Helsinki and was approved by the Joint Chinese University of Hong Kong-New Territories East Cluster Research Ethics Committee (CRE 2016.024-T). Clinical Trial Certificate was obtained from the Department of Health (Reference number: 100597). The trial was registered in clinicaltrials.gov (NCT03879889) and conducted in strict accordance with the protocol registered.

### Participants

Parents of pediatric patients were approached and screened for eligibility at the pediatric clinics and in-patient wards of the Prince of Wales Hospital (PWH), the teaching hospital of The Chinese University of Hong Kong that serves a cluster population of 1.2 million. Those with smoking habit and living with a child aged <18 years who attended services at PWH were invited. Exclusion criteria were parents with children in foster care or with unclear custody, smoking patients with pediatric, parents who had a previous allergic reaction to NRT or other relative contraindications to NRT such as cardiovascular diseases and lactation. All the participating parents provided written informed consent, and we stopped the enrollment once we achieved our target sample size.

### Intervention Group

The smoking reduction intervention included face-to-face counseling by trained research nurses and assistants on smoking reduction and NRT adherence, provision of free NRT, referral to cessation service if parents preferred, monthly phone follow-ups, and two in-person follow-up visits.

At recruitment, participants received a 20-min face-to-face individual smoking reduction counseling using the MI technique. In total, 4 weeks of nicotine patches were provided based on the participant's daily cigarette consumption. Parents who were not ready to quit immediately were encouraged to reduce cigarette consumption to at least half of their current consumption within the ensuing month; while for those who were ready to quit, cessation advice was given. Follow-ups were done monthly: week-2 (over phone), 1 month (follow-up clinic), 2 months (over phone), 3 months (over phone), 4 months (over phone), 5 months (over phone), and 6 months (clinic visit), for further counseling on smoking reduction and NRT adherence. Participants were invited to attend follow-up clinics at 1 month for receiving 4 more weeks of NRT, and at 6 months for trial effectiveness evaluation.

The intervention was designed based on the current evidence. MI is a patient-centered counseling that aims to explore and resolve ambivalence about behavioral change and has been widely adopted in smoking cessation and reduction research (30–32). This study counselors were trained by attending a workshop on smoking reduction counseling using the MI approach. To ensure the quality and fidelity, regular meetings were held every 2 weeks for case discussion, case evaluation, and continuing learning of MI. Feedbacks to improve counseling skills were provided and shared at the meetings, and we had written records of the interviews for later analyses and reviews.

Our MI was tailored by stages of change following the transtheoretical model. For the participants in the pre-contemplation stage, MI focused on the health hazards of tobacco use and ETS exposure in children, to help parents explore their motivations for smoking behavior change. For the contemplation stage, MI focused on helping parents to weigh the positive vs. negative ramifications of their smoking behavior and to resolve ambivalence. Guidance was provided to start developing plans for behavioral change. For the preparation stage, MI focused on the development of an action plan for smoking reduction, identification of potential barriers, and development of solutions. For action stage, MI focused on the progress follow-up of smoking reduction and adherence to NRT, identification of pitfalls, and modifications of action plan. For maintenance stage, MI focused on encouragement of the tobacco abstinence maintenance, identification of triggers to relapse, and development of resolutions.

Behavioral pharmacological interventions have been shown to be effective in reducing cigarette consumption (26, 33–35). Enhancement on NRT adherence was another key element of our intervention (36, 37). To increase the compliance, monthly phone follow-ups were implemented to reduce the number of hospital visits.

### Control Group

Parents were given simple brief verbal advice on smoking cessation and an information leaflet detailing currently available smoking cessation services in the community. This approach is the usual practice in our unit when we identify smoking parents. Participants were invited to attend follow-up visits at 1 month and 6 months for evaluation.

### Randomization, Blinding, and Allocation

A randomization list with block sizes of 8–12 was generated by a research staff not involved in this study. Details of the list were contained in a set of sealed, sequentially numbered envelopes. The envelopes were prepared by the same individual who generated the list and were opened by the counselors for group allocation. Each enrolled family was allocated to the next sequential number. Research personnel who performed data collection, outcome assessment, and analyses were unaware of the group allocation. However, enrolled families and counselors could not be blinded as the intervention had behavioral components, the study participants might have known whether they were in the intervention or the control group.

### Data Collection

At baseline, 1-month, 2-month, and 6-month follow-ups, data were collected using standardized questionnaires. The baseline data included demographics, medical history, smoking history, cessation stages, and parental nicotine dependence levels based on the Fagerstrom Test for Nicotine Dependence (FTND) (15, 23, 34, 38). At follow-ups, the collected data were parental cigarette consumption, FTND, and whether the participants had received any additional smoking reduction/cessation treatment during the study. At baseline and 6-month visits, urine samples of the parents and children were collected.

### Biochemical Validation and Outcome Measures

Urine cotinine is a valid biomarker of tobacco exposure and is used to validate self-reported measures (39). Moreover, a tobacco-specific alkaloid anabasine is used to distinguish pure NRT users from smokers: detectable cotinine but undetectable anabasine suggests that the participants took NRT only (39). Collected urine samples were stored at −20°C until analysis. Urinary samples were measured by ultra-performance liquid chromatography coupled online with the tandem mass spectrometer. The bioanalysis method has been validated according to US FDA Guidance for Industry on Bioanalytical Method Validation (2018) (40). The laboratory staff members were blinded to the group assignment. Individuals were considered as successful reducers if their urine cotinine level was ≤ 50% of their baseline level (34, 41), and successful quitters if their urine cotinine concentration was ≤ 115ug/L (34, 42).

Primary outcomes were: (1) urine cotinine validated parental ≥50% smoking reduction rate at 6 months; (2) self-reported parental successful smoking reduction rate at 6 months (self-reported ≥50% reduction of daily cigarette consumption compared with baseline). Secondary outcomes were (1) urine cotinine validated and self-reported parental smoking cessation rate at 6 months; (2) parental self-reported smoking reduction rate [(baseline–endline)/baseline] of cigarette consumption at 6 months; (3) change in children's urine cotinine level from baseline to 6 months.

The current RCT aimed to examine the effectiveness of a smoking reduction intervention, as it was reported that many smokers or smoking parents are not prepared to quit, and smoking reduction is an intermediate step for future complete cessation (23, 26). Therefore, we used ≥50% reduction of smoking as the primary outcomes and used smoking cessation outcomes as the secondary ones.

### Sample Size Calculation

The sample size was calculated based on the estimated differences in the self-reported ≥50% smoking reduction between intervention and control groups. Upon the protocol design, data regarding biochemically validated smoking reduction among parental smoking were limited, while self-reported outcomes have been widely used. Therefore, we adopted the self-reported smoking reduction for the sample size calculation (15). Referring to a previous local community study that examined the effectiveness of 3-visit counseling plus 4-week NRT on smoking reduction, self-reported successful reduction was 51 and 26% for the intervention and control groups, respectively (34). The sample size required to demonstrate such a difference at a 5% level of significance with 90% power, and with an assumption of a 20% dropout rate, was 105 subjects in each arm (43).

### Data Processing and Analysis

Successful smoking reduction and abstinence between groups were compared using logistic regression analyses (22, 23, 34). The comparisons between groups in the mean changes in urine cotinine levels (from baseline to 6 months), and tobacco consumption as well as FTND (from baseline to 1 month, 2 months, and 6 months) were performed using 2-way repeated measure ANOVA, while within-group differences were tested by paired Student's *t-*tests or the Mann–Whitney *U* test as appropriate. The natural log (ln)-transformations (LN) of the urine cotinine levels were performed for skewed data. In this study, there were 14 families where both parents smoked and participated, sensitivity analyses were done on one parent randomly selected from each pair.

All the analyses on self-reported outcomes were based on intention-to-treat (ITT) principle. Those lost to follow-up were counted as still smoking (unsuccessful reduction and unsuccessful cessation). For FTND scores and amount of smoking in cig/day at follow-ups, the latest available records were used if there were missing data. In addition, mixed effect models for repeated measures were adopted for fulfilling the missing data on FTND scores and amount of smoking, these additional analyses were done as sensitivity analyses. All the analyses were performed using statistical software packages SPSS (version 23.0 for Windows; SPSS Inc.). *P* < 0.05 were considered as significant.

## Results

**Figure 2:**
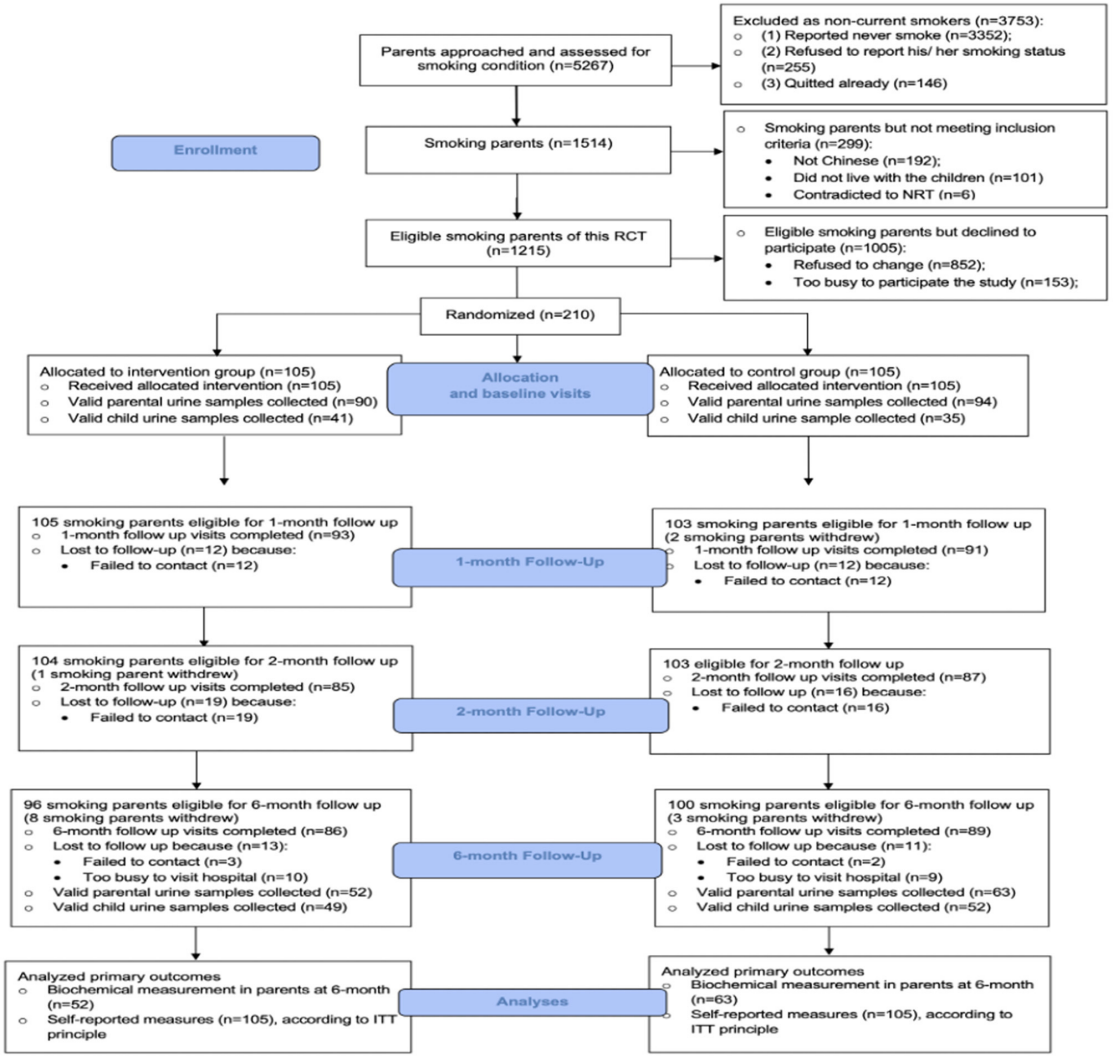
The CONSORT flow diagram.

A total of 5,267 parents were approached and screened in our pediatric units. In total, 1,514 of them were current smokers, but 299 of those parents did not meet our inclusion criteria. Among the 1,215 eligible smoking parents, 210 smoking parents (response rate: 17.3%) consented and were randomized in this RCT study. A CONSORT diagram is shown in Figure 2.

The follow-up rates were > 80% at all evaluation time-points (1 month, 2 months, and 6 months). The retention rates were similar between the two groups. No significant adverse events occurred during the study. According to the parental self-reported results, none of them (both intervention and control groups) had received additional smoking reduction/cessation intervention outside of this study protocol during the study period.

### Demographic and Clinical Characteristics

Baseline characteristics and smoking behavior of the parents are shown in Table 1. Most of the participants were middle-aged smoking fathers and were daily smokers with moderate nicotine dependence levels. The parental baseline urine cotinine concentrations were similar between groups, and the mean LN (urine cotinine concentrations) were 6.61 ± 0.90 and 6.85 ± 0.75 in the intervention and control subjects, respectively. There were no significant differences in the parental demographics and baseline smoking conditions between the two groups, except that the parents in the intervention group had a fewer number of previous quit attempts than those in the control group (*P* = 0.05).

**Table 1:** Baseline characteristics of the smoking parents (*N* = 210).

**Variables**	**All (*N* = 210)**	**Intervention (*N* = 105)**	**Control (*N* = 105)**	**P-value^**Π**^**
Age (years), mean ± SD	38.0 ± 6.9	37.4 ± 6.5	38.6 ± 7.2	0.70
Male gender, *n* (%)	172 (81.9%)	85 (81.0%)	87 (82.9%)	0.86
Both smoking parents participating, n (%)	28 (13.3%)	16 (15.2%)	12 (11.4%)	0.42
Education level				
Primary school or below, *n* (%) Secondary school, *n* (%) Tertiary education or above, *n* (%)	10 (4.8%) 149 (71.0%) 51 (24.3%)	6 (5.7%) 68 (64.8%) 31 (29.5%)	4 (3.8%) 81 (77.1%) 20 (19.0%)	0.13
Monthly household income ≤ HKD20,000, *n* (%)	72 (34.3%)	32 (30.5%)	40 (38.1%)	0.34
Overcrowding of household living place^∧^, *n* (%)	110 (52.4%)	56 (53.3%)	54 (51.4%)	0.37
Current or previous chronic medical conditions, *n* (%)	50 (23.8%)	25 (23.8%)	25 (23.8%)	1.00
Daily smoker, *n* (%)	188 (89.5%)	90 (85.7%)	98 (93.3%)	0.22
Duration of smoking more than 5 years, *n* (%)	188 (89.5%)	92 (87.6%)	96 (91.4%)	0.37
Average daily consumption in the past 1 month (cigarettes/day):				
≤ 5, *n* (%) 6–10, *n* (%) 11–20, *n* (%) 21–30, *n* (%) More than 30, *n* (%)	21 (10.0%) 63 (30.0%) 104 (49.5%) 18 (8.6%) 4 (1.9%)	12 (11.4%) 33 (31.4%) 53 (50.5%) 6 (5.7%) 1 (1.0%)	9 (8.6%) 30 (28.6%) 51 (48.6%) 11 (10.5%) 4 (3.8%)	0.28
Baseline FTND score^#^, median (IQR)	4.0 (2.0–5.0)	4.0 (2.0–5.5)	4.0 (2.0–5.0)	0.75
Had previous quit attempt(s), *n* (%)	143 (68.1%)	74 (70.5%)	69 (65.7%)	0.85
Number of previous quit attempts:				
Once, *n* (%) Twice, *n* (%) 3–5 times, *n* (%)	57 (27.1%) 47 (22.4%) 39 (18.6%)	35 (33.3%) 23 (21.9%) 16 (15.2%)	22 (21.0%) 24 (22.9%) 23 (21.9%)	0.05
Length of last quit attempt:				
<6 months, *n* (%) ≥6 months, *n* (%)	99 (47.1%) 44 (21.0%)	48 (45.7%) 26 (24.8%)	51 (48.6%) 18 (17.1%)	0.23
Motivation stage^&^				
Pre-contemplation, *n* (%) Contemplation, *n* (%) Preparation, *n* (%)	51 (24.3%) 56 (26.7%) 103 (49.0%)	31 (29.5%) 28 (26.7%) 46 (43.8%)	20 (19.0%) 28 (26.7%) 57 (54.3%)	0.12
Regular alcohol use, *n* (%)	41 (19.5%)	20 (19.0%)	21 (20.0%)	0.86
Smoking spouse, *n* (%)	49 (23.3%)	26 (24.8%)	23 (21.9%)	0.76
LN (Urine cotinine concentration)*, mean ± SD	6.73 ± 0.83	6.61 ± 0.90	6.85 ± 0.75	0.39

*Overcrowding of the living place was defined as a living space of ≤ 5.5 m^2^/person in accordance with the guideline of the Hong Kong Housing Authority*.

*^#^Fagerstrom Test for Nicotine Dependence: 1–2: low dependence; 3–4: low to moderate dependence; 5–7: moderate dependence; >8: high dependence.*

*^&^Pre-contemplation: Not intending to quit smoke in the next 6 months; Contemplation: Intending to quit in the next 6 months but not in the next 30 days; Preparation: Intending to quit in the next 30 days*.

*Valid urine samples were available for 90 parents in the intervention group and 94 parents in the control group at baseline; ^**Π**^The characteristics between intervention and control groups were compared using student's t-test, Mann–Whitney U test, and chi-square tests for parametric, non-parametric and categorical data respectively; LN: natural log (ln)-transformation.

The demographic and clinical characteristics of the children are shown in Table 2. Most of them had existing chronic medical conditions. Children's baseline demographics and urine cotinine concentrations were also similar between the two groups.

**Table 2:** Baseline characteristics of the pediatric patients (*N* = 196).

**Variables**	**All children (*N* = 196)**	**Intervention (*N* = 98)**	**Control (*N* = 98)**	***P* value^**Π**^**
Age (years), mean ± SD	5.0 ± 4.1	4.6 ± 3.8	5.2 ± 4.4	0.29
Male gender, *n* (%)	109 (55.6%)	47 (48.0%)	62 (63.2%)	0.07
Both smoking parents participating, *n* (%)	14 (7.1%)	8 (8.2%)	6 (6.1%)	0.58
Presence of siblings, *n* (%)	135 (68.9%)	66 (67.3%)	69 (70.4%)	0.33
Existing chronic medical conditions^#^, *n* (%)	182 (92.9%)	90 (91.8%)	92 (93.9%)	0.58
Existing chronic respiratory tract diseases^∧^, *n* (%)	79 (40.3%)	42 (42.9%)	37 (37.8%)	0.47
Parental perception on child's health status (Scale of 1–5)^@^, mean ± SD	3.5 ± 0.9	3.6 ± 0.9	3.5 ± 0.9	0.84
Need of long-term medication, *n* (%)	21 (10.7%)	8 (8.3%)	13 (13.5%)	0.31
Premature at birth (<37 weeks' gestation), *n* (%)	53 (27.0%)	29 (25.6%)	24 (17.9%)	0.42
Was never breastfed, *n* (%)	56 (28.6%)	28 (28.6%)	28 (28.6%)	1.00
Father smoked during child's first year of life, *n* (%)	180 (91.8%)	89 (92.7%)	91 (94.8%)	0.32
Mother smoked during child's first year of life, *n* (%)	34 (17.3%)	12 (12.5%)	22 (22.9%)	0.06
Presence of other household smoker(s), *n* (%)	28 (14.3%)	16 (16.7%)	12 (12.5%)	0.54
Have smoke ban policy at home	68 (34.7%)	32 (32.7%)	36 (36.7%)	0.55
Smoking of household smokers at home				
Sometimes, *n* (%) Always, *n* (%)	78 (40.0%) 50 (25.5%)	38 (38.8%) 26 (26.5%)	40 (40.8%) 20 (20.4%)	0.65
LN (Urine cotinine concentration + 1)*, mean ± SD	0.55 ± 0.84	0.54 ± 1.01	0.57 ± 0.58	0.92

^#^ *Including chronic respiratory tract diseases, heart disease, developmental problems, allergic rhinitis, eczema, asthma.*

*^∧^Including allergic rhinitis, asthma, chronic lung diseases*.

*^@^A Likert scale of 1–5; 1- very bad health status, 5 - very good health status*.

*Valid baseline urine samples were available for 41 children in the intervention group and 35 children in the control group.

*LN (Urine cotinine concentration + 1) was adopted as some urine samples of the children had zero values*.

*LN: natural log (ln)-transformation.*

*^**Π**^The characteristics between intervention and control groups were compared using student's t-test and the chi-square tests for continuous and categorical data, respectively*.

### Primary Outcomes

Results of urine cotinine validated outcomes are shown in Table 3. The urine cotinine validated parental successful ≥50% smoking reduction rate at 6 months in the intervention group was significantly higher than that of control: 27.1 vs. 10.0%. The intervention was shown to be effective in biochemically validated successful smoking reduction at the study end (OR = 3.34, 95% CI: 1.16–9.62, *P* = 0.02). The parental self-reported successful smoking reduction was also significantly higher in the intervention parents when compared with the control. Using ITT analysis, the self-reported successful smoking reduction rate at 6 months was 51.9% in the intervention group, which was significantly higher than the control of 20.2%, OR = 4.40, 95% CI: 2.38–8.12, *P*<*0.0*01. The intervention was shown to be effective in smoking reduction according to our primary outcome analyses.

**Table 3:** Primary and secondary outcomes.

**Outcome measures at 6-month^**∂**^**	**Intervention**	**Control**	**OR/ Mean difference^**∧**^**	***P*-value**
**Primary outcomes**				
Biochemically validated successful ≥50% smoking reduction ^∇^*, *n* (%)	13 (27.1%)	6 (10.0%)	3.34 (1.16–9.62)	**0.02**
Self-reported successful ≥50% smoking reduction, *n* (%)	55 (51.9%)	21 (20.2%)	4.40 (2.38–8.12)	**<0.001**
**Secondary outcomes**				
Biochemically validated smoking cessation ^Ω#^, *n* (%)	5 (9.2%)	1 (1.6%)	6.60 (0.75–58.36)	0.09
Parental self-reported smoking cessation (7-day point-prevalence tobacco abstinence), *n* (%)	11 (10.5%)	1 (1.0%)	12.17 (1.54–96.07)	**<0.001**
Parental self-reported reduction rate of cigarette consumption^&^(%), median (IQR)	50.0 (25.0 - 68.1)	11.3 (0.0 - 50.0)	0.2 (0.1–0.4)^∧^	**<0.001**
LN (Urine cotinine concentrations of the children at 6-month + 1)^@^, mean ± SD	0.42 ± 0.86	0.50 ± 0.73	−0.08 (−0.45–0.29)^∧^	0.68
Change in LN (Children's urinary cotinine concentrations + 1)^$&^, mean ± SD	−0.20 ± 0.68	0.09 ± 0.72	−0.06 (−0.31–0.19)^∧^	0.64
Have smoke ban policy at home, *n* (%)	51 (52.2%)	40 (40.8%)	1.57 (0.89–2.77)	0.12

^∂^*Intention to treat analyses were performed for all the self-reported outcomes*.

*^∇^48 smoking parents in the intervention group and 60 smoking parents in the control group had valid urine samples collected at both baseline and 6-month visits*.

*^Ω^52 smoking parents in the intervention group and 63 smoking parents in the control group had valid 6-month urine samples for analysis.*

*^@^49 children in the intervention and 52 in the control group had valid 6-month urine samples for analysis; LN (Urine cotinine concentration + 1) was adopted as appropriate as some urine samples of the children had zero values.*

*^$^25 children in the intervention and 21 in the control group had valid urine samples collected at both baseline visit and 6-month and were included in this analysis; LN (Urine cotinine concentration + 1) was adopted as appropriate as some urine samples of the children had zero values*.

*Individuals were considered as successful reducer if their 6-month urine cotinine level was ≤ 50% of their baseline level.

*^**#**^Individuals were considered as successful quitter if they had urinary cotinine concentration ≤ 115 ug/L at 6-month.*

*^#^Reduction rate = (baseline consumption−6-month consumption)/baseline consumption.*

*LN: natural log (ln)-transformation.*

*^∧^Mean difference. The bold value means a p-value less than 0.05: statistically significant.*

### Secondary Outcomes

Parental self-reported rate of smoking cessation (10.5 vs. 1.0%, OR = 12.17, 95% CI: 1.54–96.07, *P* < 0.001) was significantly higher in the intervention group than the control (Table 3). As for urine cotinine validated smoking cessation, only 5 out of 11 parents in the intervention group and one in the control, who had self-reported successful smoking cessation, provided urine samples for validation at both baseline and 6-month visits. All these 6 parents had validated cessation status. There were no significant differences between the two groups regarding the validated smoking cessation in the univariate (*P* = 0.09). The parental self-reported reduction rate of daily cigarette consumption at 6 months was also significantly higher in the intervention group than the control (*P* < 0.001).

In children, cotinine concentrations in the majority of the samples (97.0%) were below the detection limit of 9.23 ng/L. Children in the intervention group had lower urinary cotinine concentrations than those in the control group at 6 months, but the difference did not reach statistical significance. Changes in urine cotinine concentrations from baseline to 6 months were similar in the two groups.

### Other Outcome Measurements

The changes in parental cigarette consumption and FTND from baseline to each evaluation time point are shown in Tables 4, 5. Significantly greater reduction in both daily cigarette consumption and nicotine dependence level in the intervention group was documented throughout the study period.

**Table 4:** Parental self-reported reductions in cigarette consumption and Fagerstrom Test for Nicotine Dependence (FTND) (*N* = 210).

	**Intervention (N = 105)**	**Control (N = 105)**	**Mean difference**	***P*-value**
Parental reduction in cigarette consumption at 1-month (cig/day), median (IQR)	5.00 (1.50–8.00)	3.00 (0.00–8.25)	0.92 ((−10.71–2.38)	0.27
Parental reduction in cigarette consumption at 2-month (cig/day), median (IQR)	7.00 (2.75–10.00)	3.00 (0.00–7.00)	3.47 (1.48–5.51)	**0.001**
Parental reduction in cigarette consumption at 6-month (cig/day), median (IQR)	7.00 (3.00–10.00)	1.00 (0.00–5.00)	3.67 (1.90–5.37)	**<0.001**
Parental reduction in FTND at 1-month, median (IQR)	2.00 (0.00–3.00)	1.00 (0.00–2.00)	0.69 (0.02–1.39)	**0.05**
Parental reduction in FTND at 2-month, median (IQR)	2.00 (0.00–3.00)	1.00 (0.00–3.00)	0.69 ((−10.02–1.42)	0.06
Parental reduction in FTND at 6-month, median (IQR)	1.00 (0.00–3.00)	0.50 (−1.00–2.00)	0.95 (0.21–1.69)	**0.01**

*Reduction = Baseline condition – Follow-up condition. The bold value means a p-value less than 0.05: statistically significant*.

**Table 5:** Parental self-reported reductions in cigarette consumption and FTND (*N* = 210) (sensitivity analyses).

	**Intervention (N = 105)**	**Control (N = 105)**	**Mean difference**	***P*-value**
Parental reduction in cigarette consumption at 1-month (cig/day), median (IQR)	5.00 (0.00–8.00)	1.00 (0.00–7.00)	0.69 (−0.73–2.11)	0.34
Parental reduction in cigarette consumption at 2-month (cig/day), median (IQR)	5.00 (0.00–9.50)	2.00 (0.00–6.00)	1.89 (0.40–3.39)	**0.01**
Parental reduction in cigarette consumption at 6-month (cig/day), median (IQR)	5.00 (2.00–10.00)	0.00 (0.00–5.00)	3.25 (1.70–4.80)	**<0.001**
Parental reduction in FTND at 1-month, median (IQR)	1.00 (0.00–3.00)	0.00 (0.00–2.00)	0.60 (0.03–1.16)	**0.04**
Parental reduction in FTND at 2-month, median (IQR)	1.00 (0.00–3.00)	1.00 (0.00–2.00)	0.44 (−0.15–1.04)	0.14
Parental reduction in FTND at 6-month, median (IQR)	1.00 (0.00–3.00)	0.00 (−1.00–2.00)	0.66 (0.07–1.24)	**0.03**

*Reduction = Baseline condition – Follow-up condition. The bold value means a p-value less than 0.05: statistically significant*.

In 14 families (8 families in the intervention group and 6 families in the control group) where both smoking parents participated, sensitivity analyses on a randomly selected one yielded the same findings (Table 6).

**Table 6:** Primary and secondary outcomes (sensitivity analyses).

**Outcome measures at 6-month**	**Intervention**	**Control**	**OR/ Mean difference^∧^**	***P-*value**
**Primary outcomes**				
Biochemically validated successful ≥50% smoking reduction ^∇*^, *n* (%)	13 (28.9%)	6 (10.3%)	3.52 (1.21–10.19)	0.02
Self-reported successful ≥50% smoking reduction, *n* (%)	52 (53.6%)	20 (20.2%)	4.56 (2.43–8.59)	<0.001
**Secondary outcomes**				
Biochemically validated smoking cessation, *n* (%)^Ω#^	5 (10.2%)	1 (1.6%)	6.82 (0.77–60.44)	0.05
Parental self-reported smoking cessation (7-day point-prevalence tobacco abstinence), *n* (%)	10 (13.5%)	1 (1.3%)	11.9 (1.5–95.3)	0.004
Parental self-reported reduction rate of cigarette consumption^&^(%), median (IQR)	50.0 (25.0–76.7)	11.3 (0.0–50.0)	0.3 (0.1–0.4)^∧^	0.001

^∇^*45 smoking parents in the intervention group and 58 smoking parents in the control group had valid urine samples collected at both baseline visit and 6-month and were included in this analysis*.

*^Ω^49 smoking parents in the intervention group and 61 smoking parents in the control group had valid end-line urine samples for analysis*.

^*^Individuals were considered as successful reducer if they had 6-month urine cotinine concentrations ≤ 50% of their baseline levels.

*^**#**^Individuals were considered as successful quitter if they had urinary cotinine concentration ≤ 115 ug/L at 6-month*.

*^&^Reduction rate = (baseline consumption−6-month consumption)/ baseline consumption*.

*^∧^Mean difference. The bold value means a p-value less than 0.05: statistically significant.*

## Discussion

This study demonstrated the effectiveness of a multi-component intervention for smoking reduction in parents of patients with pediatric. One-quarter of the parents in the intervention group had biochemically validated smoking reduction and more than half had self-reported successful smoking reduction. The parental self-reported cessation rate was higher in the intervention group. However, no between-group difference was found in the urine cotinine validated cessation rate. This study has provided valuable support to the effectiveness of such intervention targeting smoking reduction for parents in clinical settings.

Both the urine cotinine validated (27.1 vs. 10.0%) and self-reported (51.9 vs. 20.2%) parental smoking reduction rates were significantly higher in the intervention group. Face-to-face individualized counseling on smoking reduction using the MI approach and free pharmacotherapy were combined in our intervention bundle. Many smokers have nicotine dependence and withdrawal symptoms are common during the cessation process (33). Pharmacotherapy helps to alleviate the symptoms and increases the chance to achieve and maintain abstinence (26, 35). A similar intervention package was adopted in a local community study for smokers who were unwilling to quit (34). The higher effectiveness in this study might be explained by the different target populations and settings that interventions are more likely to be effective when participants are recruited from healthcare settings compared to the community (33, 34). Moreover, about half of the participants recruited in this study were motivated to quit whereas the previous community study mainly recruited unmotivated smokers (34). In addition, our counseling highlighted the ETS and THS-related adverse effects on children, which might further motivate the parents to reduce tobacco use (16, 44). MI technique based on stage of change model was incorporated in our intervention (45). The technique can be easily acquired with increasing application in medical care settings (45). Counseling with a patient-centered approach encourages the participants to explore and resolve ambivalence about changing their behavior, increases self-efficacy, and helps to maintain behavioral change (30).

In this study, nearly half of the participants were in the pre-contemplation or contemplation stage at baseline, reflecting that they were not prepared to quit smoking in the next 30 days. Such barrier is common and therefore our intervention targeted reduction instead of cessation (34). An effective smoking reduction intervention is an option providing an intermediate step for complete cessation. Large (over 50%) and even moderate (25–50%) smoking reduction could increase the likelihood of future cessation (46, 47).

Nonetheless, the intervention was shown to have little impact on smoking cessation with a relatively short follow-up in this study. Although the self-reported cessation rates (10.5 vs. 1.0%) were higher in the intervention group, no statistically significant differences were found in the biochemically validated cessation rates (9.2 vs. 1.6%). Worthy to note that only less than half (5 out of 11) of the parents in the intervention group, who self-reported successful abstinence provided urine samples for analyses. According to two comprehensive reviews which aimed to explore whether smoking reduction interventions help to achieve better cessation, the average tobacco abstinence rates in the reduction intervention group was about 11%, compared with 6% in the control group (27, 28). Moreover, when compared with a previous local community study, their biochemically validated quit rates were 8.0% in the intervention group and 4.4% in the control (34). The cessation rates of our intervention group were close to the previously reported figures, while the cessation rates of the control group were much lower than expected. Smoking cessation is undoubtedly a more effective way to guarantee a smoke-free environment for children than smoking reduction. Further effort to achieve smoking cessation should be the ultimate aim of future research studies and policy development.

In this study, children's ETS exposure was validated using urine cotinine concentrations. However, the number of valid urine samples was limited as only 25.5% of the children in the intervention and 21.4% of those in the control group had valid samples collected at both baseline and the study end. The current RCT was not powered to detect the differences in children's ETS reduction between groups. Additionally, the majority of the children's urine cotinine concentrations were lower than the detection limit. Values below this level had limited accuracy and precision, casting doubt on its reliability. Children's urine cotinine concentrations in this study were relatively low when compared with other studies (48–50). One possible explanation is that most parents (87.7%) reported at the study baseline that they would keep certain distances from their children when they smoke. Further evaluation of parental smoking habits and its relation to children's ETS exposure would be needed.

Further trials with a larger sample size and longer follow-up period would be needed to evaluate the intervention's longer-term effectiveness in cessation, and its benefit on children's ETS exposure and health outcomes. The lengths of follow-up among previous similar research ranged from 1 month to 12 months, and the majority of them adopted a 6-month follow-up for their primary outcomes. Nonetheless, 6 months was relatively short and a study design that employs a longer follow-up such as 12 months would be preferred in future trials (15, 22). Being a common limitation in smoking reduction trials, our recruitment rate was moderate and was similar to previous local studies (34). This recruitment difficulty reflects that motivating smokers who are unready to change remains a major obstacle in tobacco control. For smokers who are not ready to change, smoking reduction may be an intermediate step to future cessation. However, we acknowledge the limitation of smoking reduction and in fact, smoking cessation would be the most effective way to achieve a smoke-free environment for children. Furthermore, biochemically verified smoking cessation has shown to be valid and more widely adopted outcomes than smoking reduction. Therefore, the effort to achieve parental smoking cessation would be the ultimate goal of future research. Another limitation with this study was the modest number of valid urine samples collected from smoking parents and particularly among the children subjects. Although the biochemical verification rate in this study was comparable with previous research, caution must be exercised in interpreting the biochemically validated outcomes (23, 34). In this study, the parents in the control group tended to have a higher number of previous quit attempts than those in the intervention group. However, the number of participants in each group who had previous quit attempts was similar. The exact effect of the baseline imbalance in the number of previous quit attempts on the study results was uncertain. Previous studies demonstrated that smokers who made quit attempts and failed were more likely to reduce cigarette consumption upon resumption of smoking (51). Moreover, some reported that for many smokers it could take 30 or more quit attempts before successful cessation (52, 53). There was a possibility that the parents in the control group were more likely to reduce consumption than those in the intervention group. Despite this potential limitation, this study was still able to demonstrate the effectiveness of the smoking reduction intervention.

## Conclusion

To conclude, this study demonstrated the effectiveness of a multi-component intervention in reducing tobacco consumption of smoking parents. However, effectiveness was not observed in terms of smoking cessation and reduction in children's ETS exposure. Achievement of complete smoking cessation and a smoke-free environment remains a major challenge that requires further effort and research.

## Data Availability Statement

The datasets used in the current study are available from the corresponding author upon reasonable request and with permission of The Chinese University of Hong Kong. However, restrictions apply and the data are not publicly available.

## Ethics Statement

The studies involving human participants were reviewed and approved by the Joint Chinese University of Hong Kong-New Territories East Cluster Research Ethics Committee (CRE 2016.024-T). The patients/participants provided their written informed consent to participate in this study.

## Author Contributions

SD designed the study, collected data, carried out the data analyses, drafted the initial manuscript, reviewed, and revised the manuscript. MC and RK contributed to the study design, supervised the biochemical analysis, interpreted the data, and critically reviewed the manuscript for important intellectual content. AL contributed to the study design and subject recruitment, interpreted the data, and critically reviewed the manuscript for important intellectual content. CA contributed to the study design and data analyses, interpreted the data, and critically reviewed the manuscript for important intellectual content. KC conceptualized and designed the study, recruited the subjects, supervised data collection and analyses, interpreted the data, and critically reviewed the manuscript for important intellectual content. All authors approved the final manuscript as submitted and agreed to be accountable for all aspects of the study.

## Funding

This study was supported by the Research Fellowship Scheme, Health and Medical Research Fund (Grant Number: 01150077) from the Food and Health Bureau, Hong Kong SAR, China.

## Conflict of Interest

The authors declare that the research was conducted in the absence of any commercial or financial relationships that could be construed as a potential conflict of interest.

## Publisher's Note

All claims expressed in this article are solely those of the authors and do not necessarily represent those of their affiliated organizations, or those of the publisher, the editors and the reviewers. Any product that may be evaluated in this article, or claim that may be made by its manufacturer, is not guaranteed or endorsed by the publisher.
